# Perceived organizational status and bootleg innovation: the role of moral licensing in breaking rules

**DOI:** 10.1186/s40359-025-03632-w

**Published:** 2025-12-26

**Authors:** Du-Juan Huang, Xi-Ou Gao, Wei Shao, Jun-Ru Wang

**Affiliations:** 1 College of Business Administration, Anhui University of Finance and Economics, Bengbu, Anhui Province 233030 China; 2 Chow Yei Ching School of Graduate Studies, City University of Hong Kong, Tat Chee Avenue, Kowloon, Hong Kong, 999077 China

**Keywords:** Bootleg innovation, Perceived organizational status, Moral credit, Moral credential, Intrinsic motivation

## Abstract

**Background:**

Bootleg innovation differs from traditional innovation in that it involves actions that either contravene established organizational rules and procedures or are conducted autonomously and covertly by individuals, with the aim of benefiting the organization. While prior research has highlighted a strong link between moral factors and bootleg innovation, there remains a paucity of studies exploring the moral foundations underlying such innovation. Drawing on moral licensing theory, this study examines the dual nature of bootleg innovation to elucidate the mechanism through which perceived organizational status influences employee engagement in bootleg innovation.

**Methods:**

This study constructs and verifies a moderated mediating model of organizational status perception and bootleg innovation, using exploratory factor analysis and hierarchical regression analysis based on 394 survey responses.

**Results:**

The findings indicate that: (1) Perceived organizational status has a significant positive effect on employee bootleg innovation behavior. (2) Both dimensions of moral licensing (moral credits and moral credentials) mediate the relationship between perceived organizational status and bootleg innovation behavior. (3) Intrinsic motivation positively moderates the impact of perceived organizational status on moral credits and moral credentials.

**Conclusion:**

This study offers a unique perspective on the moral dilemmas employees face in bootleg innovation, thereby providing a novel lens for understanding their bootleg innovation behavior.

## Introduction

Organizational innovation has long been framed at the intersection of fostering employee autonomy (“laissez-faire”) and enforcing regulatory oversight (“control”) [1]. While organizations seek to stimulate creativity, they also face structural constraints, such as limited resources and market competition, which necessitate screening mechanisms to ensure that new ideas align with strategic goals. However, such screening can unintentionally suppress potentially valuable innovations, prompting some employees to engage in bootleg innovation—the pursuit of innovative ideas without formal organizational approval [2]. Surveys suggest that more than 80% of companies have experienced instances of bootleg innovation [3]. As innovation activities expand, promoting practices that are both morally defensible and sustainable becomes increasingly important—particularly for innovations that challenge prevailing moral norms [4].

Existing research on bootleg innovation has primarily examined factors such as leadership styles [5–7], organizational climate [8, 9], and individual traits [10–12], providing important insights into its antecedents. Scholars have also recognized the role of employee status in shaping innovative behavior [13]. some have focused on formal status (e.g., rank, role) and its influence on resource access and tolerance for deviant actions, while others have examined informal status, such as influence, reputation, or network centrality [14], highlighting how such social capital facilitates idea implementation. Yet, most of these studies define status using external indicators—reflecting a “third-party” or structural perspective—without addressing how employees themselves subjectively perceive their standing within the organization. This externally driven approach cannot fully explain why employees with similar formal or informal positions differ in their propensity to engage in bootleg innovation. In practice, such behavior often stems from employees’ subjective assessments of their relationship with the organization—particularly whether they feel “qualified” or “valuable enough” to break rules in pursuit of change [10]. To address this gap, it is essential to examine a subjective, implicit construct closely tied to moral motivation—perceived organizational status. This is especially relevant in the Chinese context, where cultural norms emphasize hierarchy and organizational identity, and where self-perceptions are strongly linked to workplace attitudes and behaviors [15]. Perceived organizational status, defined as an individual’s sense of their standing or worth within the organization, has been linked to risk-taking, decision-making, and innovation [16, 17], but remains underexplored in the bootleg innovation literature. The present study positions perceived organizational status as a central antecedent, examining how it influences bootleg innovation through mechanisms such as moral licensing, thereby offering a novel explanation for why “good employees” might break organizational rules to innovate.

Bootleg innovation embodies a distinct moral duality: while its aim is to enhance organizational performance, reflecting a utilitarian orientation, it often involves bypassing formal norms through informal channels, thereby carrying deontological moral risks [2, 18]. Despite this moral tension, relatively few studies have examined—via a moral-psychological lens—why employees are willing to break rules to pursue such innovations. Moral licensing theory offers a valuable framework for understanding this phenomenon [19]. It operates through two primary mechanisms: moral credits, in which individuals accumulate “moral capital” from prior ethical behaviors that can later be “spent” to offset immoral acts; and moral credentials, wherein a previously established moral image redefines subsequent questionable behaviors as non-immoral [20]. Although both mechanisms produce moral licensing, their operational paths differ: moral credits imply an acknowledgement of the act’s immorality and a compensatory logic, whereas moral credentials change evaluative standards and thus blur moral boundaries. According to the theory, individuals may feel morally justified in engaging in “misconduct” when they perceive that their past “good deeds” have sufficiently elevated their moral self-image [21, 22].

Building on this logic, we propose that perceived organizational status may serve as a key psychological basis for moral licensing. Unlike approaches that infer innovative behavior from external status markers (e.g., rank, formal role, or network influence), perceived status is inherently subjective and morally charged, directly shaping employees’ evaluations of rules and their perceived license to deviate [23]. In cultural contexts such as China, which emphasize hierarchy and collective identity, high perceived status may symbolize not only access to resources and power, but also a reservoir of moral capital. Employees may construe their status—earned through position, tenure, or past contributions—as a moral investment in the organization, rationalizing deviant innovation with beliefs such as “I deserve this” or “the organization needs me.” This form of moral licensing, rooted in accumulated moral credits, enables individuals to justify rule-breaking on moral grounds [24].

Therefore, examining how employees’ subjective perceptions of organizational status trigger bootleg innovation via moral licensing addresses a key gap concerning internal psychological antecedents and also explains why otherwise morally upright individuals may engage in rule-breaking for the sake of innovation.By adopting a moral-psychological perspective, this study connects moral motivation, role identity, and innovative behavior, thereby extending the theoretical scope and practical relevance of antecedent research on bootleg innovation.

This study integrates the two mechanisms of moral licensing into the context of bootleg innovation, proposing that employees with higher perceived organizational status may accumulate moral credits or develop moral credentials based on their past contributions, thereby granting themselves psychological permission and moral justification to engage in rule-breaking innovation. This theoretical integration offers a novel lens for understanding moral judgment and motivational drivers of bootleg innovation. Identifying the conditions under which employees perceive themselves as morally licensed to break rules is critical for organizations seeking to foster innovation while maintaining ethical standards.

Assessing the morality of bootleg innovation is inherently complex. Although such behaviors violate organizational rules, they are often undertaken with the intent to benefit the organization, thereby creating ambiguity in moral evaluation. This blurred moral boundary can make it difficult for employees to judge the ethical acceptability of their actions. Moreover, individual differences in intrinsic motivation can lead to divergent moral interpretations of identical behaviors [25]. Prior research suggests that individuals with high intrinsic motivation are more inclined to seek moral justification for their actions, which further complicating moral assessments [26]. In this sense, intrinsic motivation may play a pivotal moderating role in the moral licensing process.

Grounded in moral licensing theory, this study examines the intersection of bootleg innovation and moral cognition. It develops an analytical framework that explains how high perceived organizational status can prompt employees to grant themselves greater “moral license,” thereby increasing their propensity to engage in bootleg innovation. The framework also incorporates the moderating influence of intrinsic motivation in shaping this process. By extending theoretical perspectives in organizational behavior and innovation management, the study deepens understanding of the moral underpinnings of deviant innovation and offers actionable insights for practitioners. These insights can guide managers in balancing the encouragement of innovation with the reinforcement of moral standards, ultimately supporting organizations in achieving long-term, sustainable development.

## Theoretical foundation and research hypotheses

### Perceived organizational status and bootleg innovation

Perceived organizational status refers to an employee’s subjective assessment of how they are viewed and treated by other members within the organization [27]. This perception includes several dimensions, such as the degree of respect received [28, 29], personal reputation and influence [30], and the level of organizational support and recognition obtained [31], among others. These perceptions shape employees’ psychological states and behaviors, significantly influencing their choices within the organization.

Moral licensing theory posits that individuals possess a moral self-perception, which is a self-assessment of their own moral standing [32]. Through sustained prior positive behaviors—such as helping others and contributing to the organization—individuals accumulate moral capital, thereby enhancing their moral self-perception [33]. Perceived organizational status is an informal hierarchy based on subjective evaluations by others [34, 35]. High perceived organizational status indicates that employees are highly recognized and respected by the organization, making them more inclined to proactively exhibit pro-organizational positive behaviors, such as taking initiative in change efforts, voice behaviors, and collaborative innovation [15, 16, 36], further consolidating their positive image and reputation. As positive behaviors accumulate, individuals’ moral self-perception strengthens [37]. When this perception surpasses a certain threshold, a self-regulation mechanism may be triggered, leading them to engage in immoral behaviors to restore their internal moral equilibrium [38].

Individuals with high perceived status typically enjoy greater autonomy and can better access and leverage informally available organizational resources [39–41]. This may create the illusion that they are entitled to violate organizational norms, seeing it as a privilege associated with their elevated status, thus increasing their likelihood of engaging in bootleg innovation. High perceived status, which signifies respect and influence, often positions individuals centrally within social networks, facilitating information exchange and collaboration [17]. This enhances their ability to integrate knowledge and manage innovation-related uncertainties [42], further enabling opportunities for bootleg innovation. Additionally, since bootleg innovation is conducted covertly and often remains undetected until publicly disclosed [43], individuals with high perceived status may feel they have ample justification and cover to engage in such behavior. In summary, high perceived organizational status serves dual functions: it bolsters perceived moral balance and lowers psychological barriers to engaging in bootleg innovation.

H1: Perceived organizational status has a positive impact on individuals’ bootleg innovation behavior.

### The mediating role of moral licensing in the relationship between perceived organizational status and bootleg innovation

Moral licensing theory introduces two key mechanisms: moral credits and moral credentials. Moral credits are grounded in individuals’ prior moral behaviors [33], functioning like a moral “savings account” that can be drawn upon to justify subsequent immoral actions—analogous to accumulating and spending moral capital [37]. By contrast, moral credentials provide a framework for reinterpreting morally ambiguous conduct. By leveraging the positive image established through previous good deeds, moral credentials offer individuals a “moral endorsement” when engaging in morally ambiguous actions, allowing them to avoid accusations of immorality [33, 44]. Both mechanisms involve balancing and interpreting an individual’s moral behavior, but they operate differently. Under the moral credit mechanism, the behavior is recognized as immoral and must be offset by accumulated moral assets. Conversely, the moral credential mechanism blurs the distinction between good and bad actions, enabling individuals to engage in morally ambiguous behaviors by relying on their positive historical image. This study posits that perceived organizational status may influence employees’ engagement in bootleg innovation through these two moral licensing mechanisms.

Primarily, through the moral credit mechanism, employees with high perceived organizational status may view themselves as valuable members with a strong “good reputation“ [45]. Such a positive reputation enhances moral self-perception and builds internal moral capital, thereby providing justification for potentially immoral actions [46]. Comparatively, individuals with high perceived organizational status often align with organizational values and are regarded as exemplary “good people“ [31]. This image bestows a “moral endorsement” on the individual [47], consistent with the logic of the moral credential mechanism, whereby subsequent morally ambiguous behaviors by those with high perceived status are deemed justifiable [37]. Accordingly, perceived organizational status can increase an individual’s moral credits and foster the formation of moral credentials. According to moral licensing theory, both moral credits and moral credentials increase the likelihood of interpersonal and organizational deviant behaviors [48]. When confronted with the moral dilemma posed by bootleg innovation, individuals draw on their accumulated moral credits from past organizational contributions to offset psychological burdens [49]while moral credentials help obscure the immorality of certain behaviors. This allows individuals and others to perceive morally ambiguous actions as acceptable [47], thereby protecting their reputation [50]. Additionally, moral credentials redefine immoral behavior, mitigating negative self-attributions and reducing the guilt associated with violating organizational norms [51]. It is worth emphasizing that compared to typical pro-organizational deviant behaviors (such as concealing errors or falsifying costs), bootleg innovation involves greater creativity and risk, with more complex behavioral motivations and psychological foundations [43]. First, innovation often involves breaching existing procedures and restructuring resource allocations, which requires individuals to attain an internal sense of justificatory legitimacy before making innovation decisions—that is, a belief that their rule-breaking is not merely harmless but beneficial [52]. The moral licensing mechanism serves as a crucial pathway for constructing this psychological authorization. Secondly, innovation behavior is often accompanied by the risk of failure, necessitating that individuals believe their actions are not only forgiven but also carry positive significance [53]. Moral licensing provides this basis for self-justification, enabling employees not only to believe that they are permitted to deviate but also that such deviation is warranted in the pursuit of innovation.

Thus, the following hypothesis is proposed:

H2: Perceived organizational status positively influences bootleg innovation behavior through moral licensing (a. moral credits, b. moral credentials).

### The moderating role of intrinsic motivation

Individuals’ moral perceptions are shaped by situational factors, leading to the relativity of standards in evaluating moral behavior. The extent to which positive behavior is attributed to internal factors can alter one’s recognition of moral boundaries, thereby influencing the operation of moral licensing mechanisms [24, 54]. Intrinsic motivation, which is driven by personal will and satisfaction derived from the activity itself [55], plays a critical role in this process. It helps individuals maintain a positive attitude when facing complex tasks, enhancing their sense of meaning and accomplishment in their work [55–57]. Research indicates that variations in intrinsic motivation lead to differences in moral judgment and behavior [26], making it a key moderating variable in the relationship between perceived organizational status and moral licensing mechanisms.

Employees with high intrinsic motivation tend to work autonomously and exhibit positive behaviors [58]. These spontaneous actions are perceived as morally commendable, altruistic, and sincere, in contrast to behaviors driven by external incentives, which are often seen as less morally virtuous [46]. When employees possess strong intrinsic motivation, they may attribute their enhanced perceived organizational status to their exceptional contributions. This reinforces their moral self-image [26], with studies showing that this impacts their sense of moral licensing [59]. Individuals with a positive organizational image are often labeled as “morally good“ [47]. Their perceived status is closely linked to this idealized moral image, which significantly boosts their self-perceived morality and helps them accumulate moral capital to justify future immoral behavior, facilitating moral licensing.

Conversely, employees with low intrinsic motivation are less likely to attribute improvements in their status to internal factors. In these cases, they may attribute their perceived status enhancement to external pressures, obligations, or incentives [60], rather than to their moral or altruistic actions. The absence of a moral connection to their behavior hinders the reinforcement of their moral self-image, limiting their ability to accumulate moral capital and making it difficult to form moral licensing [46]. Consequently, low intrinsic motivation slows or even halts the process of constructing moral licensing based on perceived organizational status. Even if employees perceive an improvement in their status, they may not gain enough moral credit to support subsequent immoral behavior.

Thus, the following hypothesis is proposed:

*H3a:* Intrinsic motivation positively moderates the relationship between perceived organizational status and moral licensing (a. moral credits, b. moral credentials). Specifically, the higher the level of intrinsic motivation, the stronger the positive relationship between perceived organizational status and moral licensing; conversely, the lower the level of intrinsic motivation, the weaker the relationship.

Positive behaviors driven by intrinsic motivation may increase employees’ propensity to engage in morally ambiguous actions [26]. Building on hypotheses H2 and H3a, this study further predicts that the effect of perceived organizational status on bootleg innovation through moral licensing (a. moral credits, b. moral credentials) is moderated by the level of intrinsic motivation, indicating a moderated mediation effect.

**Fig. 1:**
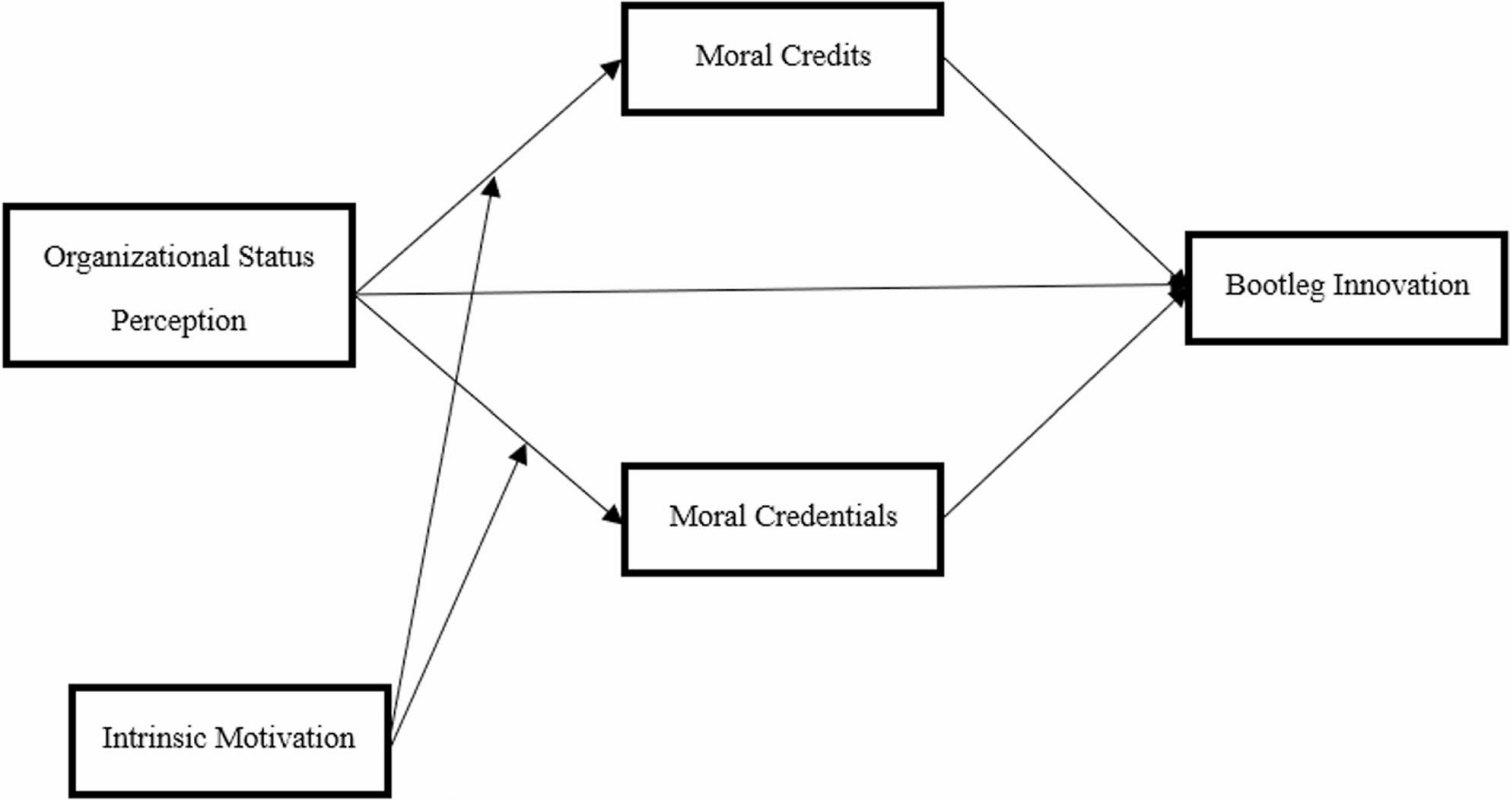
Theoretical model

*H3b:* Intrinsic motivation moderates the indirect effect of perceived organizational status on bootleg innovation through moral licensing (a. moral credits, b. moral credentials). When employees have a high level of intrinsic motivation, the mediating effect of moral licensing is strengthened. In summary, the conceptual model of this study is shown in Fig. 1.

## Methodology

In this section, we will introduce the research sample and data, variable measurements, and the analysis procedures we adopted.

### Sample and data

Data for this study were collected through a questionnaire survey of employees from 31 innovative enterprises located in regions including Shanghai, Suzhou, Nantong, Hangzhou, and Ningbo. In this study, “innovative enterprises” were identified based on government-issued recognition lists in China (e.g., provincial or municipal lists of “Innovative Enterprises,” “High-tech Enterprises,” or “Science & Technology Little Giants”). Given anonymity and data access constraints, we adopted a self-report-based screening approach: each respondent was asked to confirm whether their company had received such recognition within the past three years, and whether it had a formal R&D/technical/design function or had undertaken new product/process/critical workflow innovation in the same period. For part of the sample, this confirmation was followed by random back-checks. This operationalization aligns with the conceptual definition while remaining practically feasible under our study’s constraints. To preserve the anonymity of the participating companies, specific names are not disclosed in the manuscript. Sample sources were verified with selected participants during the follow-up phase to ensure data validity and industry representativeness.

To enhance sample representativeness and data reliability, three distinct questionnaire distribution methods were employed: (1) Offline enterprise surveys: leveraging MBA program resources from a university in Anhui, enterprises employing program participants were selected as survey sites. With the assistance of these MBA participants, on-site surveys were conducted among design and technical department employees, with researchers guiding respondents to ensure questionnaire quality. A total of 96 questionnaires were collected in this stage, with 81 valid after excluding invalid responses, yielding an effective response rate of 84.38%; (2) Alumni-assisted surveys: via alumni associations and personal networks, trained alumni were entrusted to distribute paper questionnaires within their respective organizations and organize responses. This method yielded 180 returned questionnaires, with 166 deemed valid after screening for logical consistency and missing data, resulting in a valid response rate of 92.22%; (3) Third-party platform collection: a professional survey agency was commissioned to distribute online questionnaires to selected respondents filtered by industry and job role. Data quality was controlled through restrictions on response duration, randomization of question order, and consistency checks. From 200 returned questionnaires, 147 were retained as valid, corresponding to a valid response rate of 73.50%. The questionnaires administered through the three distribution channels were identical in content, item order, and rating scale. To evaluate potential heterogeneity, we conducted mean-difference test on the main variables—including perceived organizational status and bootleg innovation—across the three subsamples. No significant differences were found, indicating good comparability of the data obtained via different channels. Consequently, all valid responses were pooled for unified model estimation.

The questionnaire survey spanned six months, resulting in 476 returned questionnaires and an overall valid response rate of 82.78%. Except for control variables, all scales in this paper are rated on a Likert-5 scale, with 1 indicating “completely disagree” and 5 indicating “completely agree”. The demographic characteristics of the final sample are shown in Table 1.

**Table 1:** Characteristics of demographic variables

	Category	Number of Respondents	Percentage (%)
Gender	Male	187	47.5
	Female	207	52.5
Age Group	25 years and below	77	19.5
	26–35 years	172	43.7
	36–45 years	108	27.4
	46 years and above	37	9.4
Education	College degree or below	152	38.6
	Bachelor’s degree	202	51.3
	Master’s degree or above	40	10.2
Work Experience	Less than 1 year	26	6.6
	1–3 years	82	20.8
	3–5 years	168	42.6
	More than 5 years	118	29.9
Position	Grassroots manager	301	76.4
	Middle manager	75	19
	Senior manager	18	4.6

### Variable measurement

The study employed scales from authoritative journals both domestically and internationally, strictly adhering to the “translation-back-translation” procedure as proposed by Brislin (1970) [61], ensuring their validity in the local survey environment. All items were measured using a 5-point Likert scale.

1. Organizational Status Perception: Referring to the organizational status perception scales by Shan Hongmei (2015) [62] and Eisenberger (2002) [63]. An example item is “Organizational decisions often seek my opinions and suggestions.” The Cronbach’s α coefficient for this scale was 0.859.
2. Moral Credits: Using a 5-item scale from Lin (2016) [64] and others. An example item is “Good behavior can accumulate my moral reputation.” The Cronbach’s α coefficient for this scale was 0.876.
3. Moral Credentials: Adopting a 5-item scale from Aquino (2002) [25] and others. Participants first read about nine positive moral traits (caring, compassion, fairness, friendliness, generosity, helpfulness, diligence, honesty, kindness) and then assessed their alignment with these traits. An example item is “These qualities are not important to me.” The Cronbach’s α coefficient for this scale was 0.878.
4. Bootleg Innovation: Using a 5-item scale from Crisuolo (2014) [2] and others. An example item is “I voluntarily spend time on unofficial projects to enrich future official projects.” The Cronbach’s α coefficient for this scale was 0.871.
5. Intrinsic Motivation: Referring to the intrinsic motivation scales by Wang Bin (2007) [65] and Amabile (1994) [66], originally developed as a 6-item scale by Amabile (1994) [66]. An example item is “I feel more joyful when I can set my own goals.” The Cronbach’s α coefficient for this scale was 0.908.
6. Control Variables: Drawing on prior research related to perceived organizational status and deviant innovation behaviors [10, 14, 15, 67], this study controlled for employee gender, age, education level, position level, and years of work experience, all of which are relevant factors to bootleg innovation [68]. These variables may influence individual innovation behavior through multiple mechanisms. For example, gender and age can affect risk propensity and moral judgment tendencies, thereby impacting the likelihood of engaging in bootleg innovation. Educational background and tenure relate to employees’ knowledge reserves and degree of autonomy within the organization, potentially affecting both innovative capability and willingness. Accordingly, these demographic variables were included as control factors in the regression analysis to more accurately assess the causal pathways among the primary variables.

### Analysis procedures

This study primarily utilized SPSS 24.0, AMOS 24.0, and the PROCESS macro for data analysis. After conducting confirmatory factor analysis (CFA) using AMOS 24.0 to validate the research variables, descriptive statistics, correlation analysis, and hypothesis testing were performed using SPSS 24.0. Additionally, Hayes’ PROCESS macro was employed for secondary validation of the mediating effects and testing moderated mediation effects.

### Empirical analysis

#### Common method bias test

Following Podsakoff et al. (1986) [69] and Du et al. (2012) [70], Harman’s single-factor test was conducted to examine common method bias. The maximum factor variance explained was 34.165%, which is less than the critical threshold of 40%. This indicates that there is no significant issue of common method bias in this study. Additionally, following Zhou Hao and Long Lirong (2004) [71], we employed the unmeasured latent method factor approach by introducing a common method factor as a latent variable into the baseline model and comparing model fit before and after its inclusion. The analysis revealed that controlling for the common method factor did not significantly improve model fit (χ²/df = 1.055, RMSEA = 0.012, CFI = 0.998, TLI = 0.997), suggesting that common method bias does not significantly affect the five main variables in this study.

#### Confirmatory factor analysis

The results of the confirmatory factor analysis are shown in Table 2. The five-factor model demonstrated the best fit, indicating good convergent validity of the construct measurements. The fit indices for the other six models showed poorer fit with the observed data, as evidenced by higher chi-square values and lower model fit indices, confirming that the hypothesized model outperformed alternative models.

**Table 2:** Confirmatory factor analysis

Model	χ2	df	χ2/df	RMSEA	CFI	TLI
Five-factor model(Mr, Mc, Bi, Pos, Im)	281.911	265	1.064	0.013	0.997	0.997
Four-factor model(Mr + Mc, Bi, Pos, Im)	975.182	269	3.625	0.081	0.875	0.861
Three-factor model(Mr + Mc, Bi, Pos + Im)	1512.940	272	5.562	0.106	0.781	0.758
Two-factor model(Mr + Mc + Bi, Pos + Im)	2107.126	274	7.690	0.129	0.676	0.645
Single-factor model(Mr + Mc + Bi + Pos + Im)	2930.625	275	10.657	0.155	0.531	0.488

In Table 2, *Mr* refers to Moral credits (or reputation), *Mc* refers to Moral credentials, *Bi* refers to Bootleg innovation, *Pos* refers to Perceived organizational status, and *Im* refers to Intrinsic motivation

#### Descriptive statistics and correlational analysis

The average values, variances, and results of the correlation analysis for each variable are shown in Table 4. The data indicate significant positive correlations between perceived organizational status and moral credit (*r* = 0.346, *p* < 0.01), moral credential (*r* = 0.371, *p* < 0.01), and bootleg innovation (*r* = 0.396, *p* < 0.01). Moral credit (*r* = 0.314, *p* < 0.01) and moral credential (*r* = 0.403, *p* < 0.01) also show significant positive correlations with bootleg innovation. All hypotheses of this study have been preliminarily validated.

**Table 3:** Correlation analysis result

Variables	Mean	Standard Deviation	1	2	3	4	5	6	7	8	9	10
Gender	1.530	0.500										
Ag	2.270	0.881	0.039									
El	1.720	0.638	−0.025	−0.032								
Ywe	2.960	0.879	0.049	0.862**	0.002							
Jp	1.280	0.543	0.053	0.444**	−0.099	0.366**						
Mr	3.269	0.936	0.081	−0.061	0.037	−0.042	0.015	(0.876)				
Mc	3.283	0.945	0.034	0.048	0.010	0.044	−0.007	0.388**	(0.878)			
Pos	3.357	0.958	0.003	0.001	0.004	−0.008	0.018	0.346**	0.371**	(0.859)		
Bi	3.286	0.923	−0.035	−0.022	−0.016	0.019	−0.018	0.314**	0.403**	0.396**	(0.871)	
Im motivation	3.290	0.992	0.029	0.024	0.100*	−0.003	0.060	0.426**	0.381**	0.432**	0.426**	(0.908)

In Tables 3 and 4, and 5, *Gr* Refers to Gender, *Ag* Refers to Age group, *El* Refers to Educational level, *Ywe* Refers to Years of work experience, *Jp* Refers to Job position, *Mr *Refers to Moral reputation (Moral credits), *Mc* Refers to Moral credentials, *Bi *Refers to Bootleg innovation, *Pos* Refers to Perceived organizational status, and *Im* Refers to Intrinsic motivation

same below; values in parentheses are Cronbach’s alpha coefficients for each variable; sample size *N* = 394, same below

*p < 0.05

**p < 0.01

***p < 0.001

### Main effects and mediation analysis

The study utilized SPSS 24.0 software and employed hierarchical regression analysis to examine the main effects and mediation effects(as shown in Table 4). After controlling for variables such as gender, age, and education, the addition of perceived organizational status in Model M6 revealed a significant positive correlation with bootleg innovation (*r* = 0.383, *p* < 0.001). This finding suggests that perceived organizational status significantly promotes bootleg innovation when accounting for other influencing factors, thereby validating Hypothesis 1.

In Models M2 and M4, by including perceived organizational status as an independent variable based on Models M1 and M3, perceived organizational status positively correlated with moral credit (*r* = 0.337, *p* < 0.001) and moral credential (*r* = 0.367, *p* < 0.001), respectively. Additionally, in Model M6, after incorporating mediator variables such as moral credit and moral credential, Models M7 and M8 demonstrated significant positive correlations with bootleg innovation (moral credit: *r* = 0.203, *p* < 0.001; moral credential: *r* = 0.292, *p* < 0.001). According to Model M9, the coefficient of perceived organizational status gradually decreased (from *r* = 0.383 to *r* = 0.248, *p* < 0.001) with the inclusion of mediator variables, indicating significant mediation effects of moral credit and moral credential on the relationship between perceived organizational status and employee bootleg innovation. Thus, Hypothesis 2 is supported.

**Table 4:** Test results of the main effect and the mediating effect

Variables	Mr		Mc		Bi				
	M1	M2	M3	M4	M5	M6	M7	M8	M9
(Constant)	2.939	1.857	3.084	1.860	3.341	2.061	1.684	1.517	1.355
Control variables									
Gr	0.154	0.152	0.063	0.061	−0.068	−0.070	−0.101	−0.088	−0.104
Ag	−0.124	−0.132	0.063	0.055	−0.157	−0.166	−0.140	−0.182	−0.164
El	0.060	0.056	0.014	0.010	−0.032	−0.036	−0.048	−0.039	−0.046
Ywe	0.037	0.050	0.005	0.018	0.161	0.175	0.165	0.170	0.165
Jp	0.092	0.079	−0.062	−0.075	−0.013	−0.027	−0.043	−0.005	−0.018
Independent variable									
Pos		0.337***		0.367***		0.383***	0.315***	0.276***	0.248***
Mediating variable									
Mr							0.203***		0.125*
Mc								0.292***	0.255***
F	1.147	9.974***	0.351	10.722***	0.629	12.868***	14.036***	17.724***	16.550***
ΔF		53.337***		62.303***		73.476***	17.710***	49.234***	
R2	0.015	0.134	0.004	0.143	0.008	0.166	0.203	0.243	0.256
ΔR2		0.119		0.138		0.158	0.037	0.077	

To further verify the robustness of the dual mediation effect, Hayes’ PROCESS macro was employed for secondary validation of the mediating effects of moral credit and moral credential. Bootstrap was set to 5000 times to run for the mediating effect test, with a 95% confidence interval. Results indicate that mediating effect of perceived organizational status through moral reputation then to bootleg innovation is 0.042, CI= [0.008, 0.080]. The mediating effect of perceived organizational status through moral credential then to bootleg innovation is 0.093, CI= [0.054, 0.139]. Therefore, hypothesis 2 is further validated.

#### Moderation effect testing

The moderation effect testing of intrinsic motivation is shown in Table 5. The interaction terms between perceived organizational status and intrinsic motivation were pre-centered to avoid multi-collinearity. Model M12 indicates a significant positive moderating effect of the interaction between perceived organizational status and intrinsic motivation on moral credit (*r* = 0.214, *p* < 0.001). Model M15 demonstrates a significant positive moderating effect of the interaction between perceived organizational status and intrinsic motivation on moral credential (*r* = 0.256, *p* < 0.001). Hypothesis 3a is supported.

**Table 5:** Test results of moderating effect

Variables	(Mr)Moral reputation or credit			(Mc)Moral credential		
	M10	M11	M12	M13	M14	M15
(Constant)	2.984	1.413	1.525	3.084	1.494	1.629
Control variables						
Gr	0.154	0.134	0.117	0.063	0.046	0.025
Ag	−0.124	−0.159	−0.153	0.063	0.032	0.039
El	0.060	0.003	−0.018	0.014	−0.034	−0.058
Ywe	0.037	0.082	0.061	0.005	0.045	0.019
Jp	0.092	0.044	0.084	−0.062	−0.104	−0.057
Independent variable						
Pos		0.195***	0.165*		0.249***	0.214***
Moderator variables and interaction terms						
Im		0.320***	0.308***		0.264***	0.249***
Pos*Im			0.214***			0.256***
F	1.147	16.081***	17.832***	0.351	14.095***	17.484***
ΔF		52.653***	23.520***		48.243***	33.019***
R2	0.015	0.226	0.270	0.004	0.204	0.266
ΔR2		0.211	0.045		0.199	0.063

**Fig. 2:**
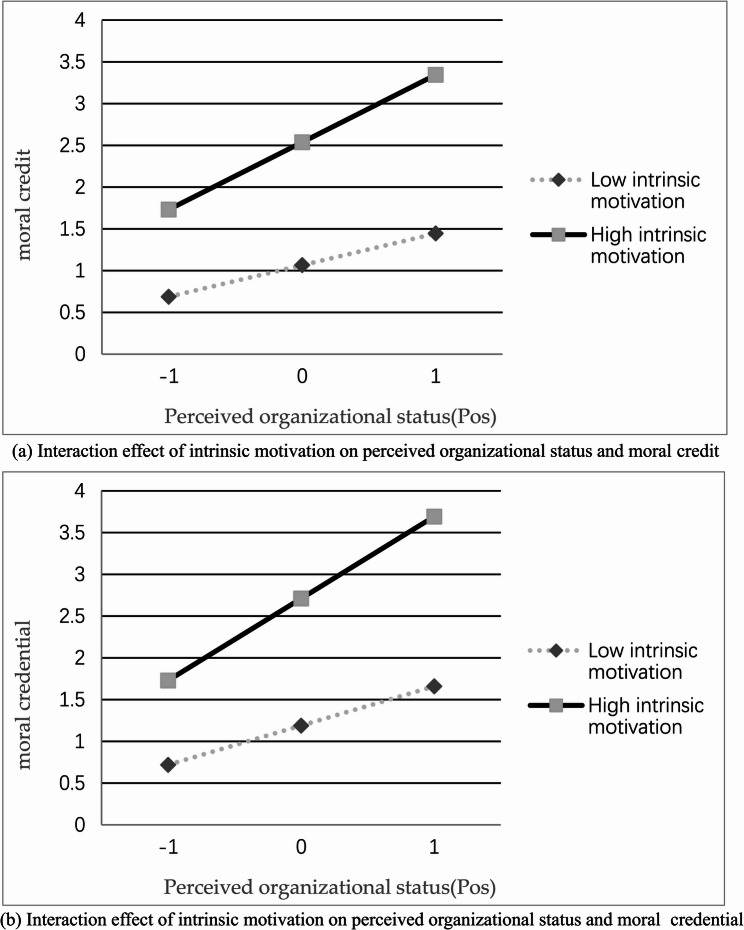
Moderating effect of intrinsic motivation. **a** Interaction effect of intrinsic motivation on perceived organizational status and moral credit. **b** Interaction effect of intrinsic motivation on perceived organizational status and moral credential

To provide a more intuitive illustration of the moderating effects of intrinsic motivation on the relationship between employees’ perceived organizational status and moral credit as well as moral credential, moderation effect plots were generated (see Fig. 2a and b). From Fig. 2, it is evident that as intrinsic motivation levels increase, perceived organizational status enhances both moral credit and moral credential in a positive and reinforcing manner.

### Testing for moderated mediation

For the moderated mediation hypothesis (Hypothesis 3b), this study used Model 7 from the PROCESS macro to verify it. According to the results presented in Table 6., on the first path, there is a significant difference in the indirect effect of perceived organizational status on bootleg innovation through moral credit at different levels of intrinsic motivation(index = 0.027, 95% confidence interval [0.005, 0.052], not including 0). Specifically, at low levels of intrinsic motivation, the indirect effect of organizational status on bootleg innovation through moral credit is not significant (indirect effect = −0.006, confidence interval [−0.027, 0.010], including 0). However, at high levels of intrinsic motivation, the effect is significant (indirect effect = 0.047, confidence interval [0.004, 0.014], not including 0). This indicates that higher levels of intrinsic motivation strengthen the mediating role of moral credit, thus demonstrating moderated mediation. Furthermore, the indirect effect difference between high and low groups is significant (indirect effect = 0.053, confidence interval [0.010, 0.103], not including 0).

On the second path, there is also a significant difference in the indirect effect of perceived organizational status on bootleg innovation through moral credential at different levels of intrinsic motivation (index = 0.065, 95% confidence interval [0.036, 0.101], not including 0). At low levels of intrinsic motivation, the indirect effect of organizational status on bootleg innovation through moral credential is not significant (indirect effect = −0.010, confidence interval [−0.046, 0.023], including 0). Conversely, at high levels of intrinsic motivation, the effect is significant (indirect effect = 0.119, confidence interval [0.069, 0.174], not including 0). This indicates that higher levels of intrinsic motivation enhance the mediating role of moral credential, thus confirming moderated mediation. Moreover, the indirect effect difference between high and low groups is significant (indirect effect = 0.130, confidence interval [0.071, 0.200], not including 0). In conclusion, intrinsic motivation significantly moderates the mediated effect of moral credit and moral credential on bootleg innovation through perceived organizational status, confirming Hypothesis 3b.

**Table 6.** Test results of moderated mediation

Mediating variable	Mediator			Index of moderated mediation	
	Moderator	Effect	(CI)	Index	(CI)
(Mr)Moral reputation(credit)	Low intrinsic motivation	−0.006	[−0.027,0.010]	0.027	[0.005,0.052]
	High intrinsic motivation	0.047	[0.004,0.014]		
	The difference between groups (high - low)	0.053	[0.010,0.103]		
(Mc)Moral credential	Low intrinsic motivation	−0.010	[−0.046,0.023]	0.065	[0.036,0.101]
	High intrinsic motivation	0.119	[0.069,0.174]		
	The difference between groups (high - low)	0.130	[0.071,0.200]		

## Discussion

This study examines the impact of perceived organizational status on bootleg innovation through the lenses of moral licensing and intrinsic motivation. Based on empirical analysis of 394 valid data samples, the study reaches several key conclusions. First, perceived organizational status has a significant positive effect on employees’ bootleg innovation behaviors, aligning with findings in the existing literature. For instance, prior research suggests that employees with high perceived status tend to exhibit greater autonomy and innovation [17]. However, this study extends the scope of previous research by introducing bootleg innovation as a unique form of innovative behavior. Traditionally, high perceived status has been seen as a driver of positive, rule-compliant behaviors within organizations. This study, however, reveals that perceived status can also promote innovation through non-compliant or bootleg means. This finding enriches the understanding of informal innovation behaviors in organizations and highlights the dual nature of perceived organizational status.

Second, moral licensing acts as a mediating factor between perceived organizational status and bootleg innovation. By accumulating moral credits or constructing moral credentials, employees can find moral justifications for engaging in bootleg behaviors. This finding supports the moral licensing theory proposed by Miller and Effron(2010), which posits that an individual’s past moral actions can grant “moral license” for future immoral behaviors [33]. The innovation of this study lies in applying this theory to the organizational context, clearly identifying the mechanisms through which moral licensing influences bootleg innovation. This finding also resonates with prior work linking employees’ moral behavior to organizational outcomes, underscoring the critical role of moral licensing in both facilitating and constraining innovation [10].

Third, the study reveals that intrinsic motivation positively moderates the relationship between perceived organizational status and moral licensing mechanisms. Specifically, when employees have high intrinsic motivation, the influence of perceived status on moral credits and moral credentials is significantly amplified. This finding is consistent with Deci and Ryan’s (2000) self-determination theory, which argues that individuals with high intrinsic motivation are more likely to internalize external status and achievements into personal values and behavior [72]. By incorporating intrinsic motivation into the analysis, this study deepens the understanding of how employees with varying levels of motivation use perceived status and moral licensing to rationalize bootleg behaviors.

Particularly, when employees possess strong intrinsic motivation, they are more inclined to view their elevated status as a result of their contributions to the organization, thus providing moral justification for their bootleg innovation. This aligns with Lammers et al. (2010), who suggest that high-power individuals tend to use moral licensing mechanisms to free themselves from behavioral constraints [73]. Therefore, this study not only extends the application of moral licensing theory but also offers theoretical insights for managers to identify and manage bootleg innovation among high-status employees in practice.

From a cultural perspective, this study was conducted in China, a context characterized by high power distance, collectivism, and strong emphasis on role responsibility [74]. These cultural traits may amplify the psychological mechanisms found in this study. For instance, in a collectivist environment, high-status employees may feel stronger moral obligations toward organizational goals, leading to moral justifications for unauthorized innovation. Similarly, traditional Chinese notions such as merit offsetting fault or tolerance of loyal deviance may make bootleg innovation appear more acceptable if it serves the organization’s interests. These culturally embedded factors underscore the need for further cross-cultural validation of the proposed model.

### Theoretical significance

First, this study significantly expands the theory of bootleg innovation by addressing the gap in the literature concerning the moral dilemmas it creates. By integrating perspectives from organizational behavior and moral psychology, this study applies moral licensing theory to the bootleg innovation context and distinguishes two mediating mechanisms—moral credits and moral credentials—within the model. It also examines the moderating effect of intrinsic motivation. This approach uncovers the underlying mechanisms linking perceived organizational status and bootleg innovation. Departing from works that emphasize individual psychology, leadership, or organizational culture, we show that bootleg innovation should be understood not merely as a trade-off between compliance and deviation but also as a moral tension between utilitarian considerations and deontological norms. By introducing concepts from moral psychology, this research both addresses scholarly calls for multi-faceted examinations of deviant innovation triggers [75] and opens a new theoretical avenue for understanding bootleg innovation. It further deepens our understanding of its driving forces and provides a solid foundation for future research. Future studies could further explore the complex causes and implications of bootleg innovation from a moral perspective.

Second, this research enriches the understanding of the consequences of perceived organizational status, revealing how individual perceptions of status deeply influence behavioral choices within complex social interactions and hierarchical structures. Whereas prior research has emphasized positive outcomes of perceived status—e.g., voice behavior [76], innovation performance [15], and organizational citizenship behavior [77] —our findings indicate that perceived status also expands access to resources and influence while blurring organizational normative boundaries, thereby increasing the likelihood of deviant behaviors. Notably, this study challenges the traditional view that higher perceived status always leads to positive organizational outcomes. Instead, our findings suggest that increased perceived status may encourage engagement in deviant behaviors. This discovery offers a novel perspective on the negative consequences of perceived status and prompts a reevaluation of the role of power and status within organizations.

Third, the study shows how perceived organizational status influences individuals’ moral judgments and can provide psychological permission to engage in bootleg innovation, thereby extending the application of moral licensing theory. Diverging from prior research grounded in face theory [14], which examines how employees’ organizational identity and status characteristics influence face-related pressure perceptions and subsequently affect deviant innovation, our study innovatively employs moral licensing theory to investigate how varying levels of perceived organizational status affect moral self-perception, which in turn influences employees’ decisions to engage in bootleg innovation. This distinction clarifies alternative psychological pathways through which perceived status affects deviant innovation and broadens the theoretical application of moral licensing. This finding aligns with the work of Klotz et al. (2013), who emphasized the importance of self-perception in immoral behavior [46]. Our research clarifies the individual moral decision-making process and highlights the organizational contexts in which individuals draw on moral resources to rationalize rule-breaking. The study distinguishes between the two mechanisms of moral licensing—moral credits and moral credentials—highlighting their distinct roles: on the one hand, perceived organizational status helps individuals accumulate moral credits, which serve as “moral capital” to justify immoral actions; on the other hand, it shapes a positive self-image, providing a “moral endorsement” for engaging in morally ambiguous behaviors. This distinction refines prior work that conflated the two mechanisms and enriches theoretical accounts of moral licensing. Moreover, by empirically testing boundary conditions, the study responds to calls to identify factors that trigger moral licensing effects and offers substantive theoretical insights.

### Practical implications

First, organizational managers should adopt a rational attitude toward employees’ bootleg innovation behaviors, avoiding overly aggressive or rigid measures to prohibit such actions, as such measures may escalate conflicts and lead to adverse outcomes. When necessary, managers can grant employees with high creativity and in significant organizational positions a certain degree of autonomy, allowing them to pursue unplanned innovative ideas. This approach can effectively guide employees toward engaging in productive bootleg innovation.

Second, organizations should strengthen the construction of moral cultures, clearly defining moral standards and continuously enhancing employees’ moral awareness through ongoing ethics education. Managers should establish clear mechanisms for moral evaluation, regularly assessing employee behavior to prevent the misuse of past “good deeds” to justify future immoral actions. By setting explicit moral guidelines and providing regular moral feedback, organizations can mitigate the negative behaviors that may arise from moral licensing.

Third, organizations should design work environments that stimulate and sustain employees’ intrinsic motivation. For example, assigning meaningful and challenging tasks and offering opportunities for personal growth can enhance employees’ internal drive. This intrinsic motivation encourages employees with high perceived organizational status to engage in unconventional and innovative behaviors, contributing more spontaneously to the organization’s success. At the same time, managers should ensure that the autonomy provided is accompanied by clear value guidance, ensuring that employees’ innovation efforts are aligned with the organization’s long-term goals.

### Future research directions

Firstly, due to the regional bias in sample distribution, there may be skewness in the data. More importantly, this study employs a cross-sectional survey design, which, while capable of revealing associations between variables, is limited in establishing clear causal inferences. Future research should adopt longitudinal tracking designs or experimental methods to strengthen causal identification and validation, thereby enhancing the scientific rigor and explanatory power of findings. Additionally, researchers could further expand the sample scope and utilize multiple survey approaches to improve data representativeness and reliability. Secondly, the variables selected do not cover firm-level factors, such as organizational culture and team atmosphere, which are potential directions for future research expansion. Furthermore, the moderating variable in this study—intrinsic motivation—reflects an individual-level internal factor with relatively limited practical manipulability. Future studies might introduce more context-sensitive and managerially actionable variables, such as organizational incentives or leadership empowerment, to increase the practical relevance of the findings. Lastly, the strength of moral licensing effects is constrained by individual personality traits, which are influenced by cultural backgrounds. Therefore, scholars are encouraged to adopt a cross-cultural perspective to explore moral licensing effects across different cultural contexts.

## Data Availability

The raw data supporting the conclusions of this article will be made available by the authors, without undue reservation, to any qualified researcher.
